# Retraction Note to: Downregulation of miR-483-5p decreases hypoxia-induced injury in human cardiomyocytes by targeting MAPK3

**DOI:** 10.1186/s11658-021-00252-1

**Published:** 2021-03-05

**Authors:** Yan Hao, Haitao Yuan, Houzhi Yu

**Affiliations:** Department of Cardiology, Shandong Provincial Hospital Affiliated To Shandong University, 324 Jingwu Road, Jinan, 250021 Shandong China

## Retraction to: *Hao et al. Cellular & Molecular Biology Letters* (2020) 25:20 https://doi.org/10.1186/s11658-020-00213-0

The authors have retracted this article because they found that some of the published data related to Fig. 5c were biased or even reversed in subsequent repeated experiments.

All authors agree to this retraction.

